# Baicalin Induces Gastric Cancer Cell Pyroptosis through the NF-κB-NLRP3 Signaling Axis

**DOI:** 10.7150/jca.89986

**Published:** 2024-01-01

**Authors:** Jiatong Liu, Xiafei Qi, Peixing Gu, Liuxiang Wang, Siyuan Song, Peng Shu

**Affiliations:** 1 Affiliated Hospital of Nanjing University of Chinese Medicine, Jiangsu, Nanjing, 210029, China.; 2 Nanjing University of Chinese Medicine, Jiangsu, Nanjing, 210029, China.; 3 Jiangsu Provincial Hospital of Chinese Medicine, Jiangsu, Nanjing, 210029, China.

**Keywords:** Gastric cancer, Baicalein, Pyroptosis, Traditional Chinese medicine, NLRP3

## Abstract

Pyroptosis, a highly regulated form of cell death, could hold the key to revolutionizing cancer treatment. With cancer posing a significant global health challenge due to its high morbidity and mortality rates, exploring unconventional therapeutic approaches becomes imperative. Chinese medicine, renowned for its holistic principles, presents intriguing possibilities for treating gastric cancer (GC). Notably, baicalin, a prominent component found in the traditional Chinese herb Scutellaria baicalensis Georgi, has shown promising clinical potential in gastric cancer treatment.To shed light on this intriguing phenomenon, a multidisciplinary approach was undertaken, combining systems biology, bioinformatics, and *in vitro* studies. The primary objective was to unravel the intricate workings underlying baicalein's ability to promote gastric cancer cell pyroptosis.The findings from this comprehensive study unveiled an essential signaling axis involving NF-κB-NLRP3, which plays a pivotal role in the process of baicalein-induced pyroptosis in gastric cancer cells. As the investigation progressed, it became evident that baicalein exhibited a remarkable capability to reverse the effects of the NLRP3 inhibitor, MCC950 Sodium. Excitingly, the efficacy of cell pyroptosis induction by baicalein demonstrated a discernible dose-dependent relationship, showcasing its potential as a valuable therapeutic agent.The complex nature of these findings underscores the intricate interplay between baicalein, NF-κB-NLRP3 signaling, and gastric cancer cell pyroptosis. As the scientific community delves deeper into the world of Pyroptosis and its therapeutic implications, baicalein's potential as a game-changer in the fight against gastric cancer becomes increasingly evident.

## Introduction

Gastric cancer, which accounts for a significant portion (approximately 6%) of global cancer cases 1, continues to afflict East Asia while demonstrating a declining incidence in the Western Hemisphere 2. Although early surgical intervention offers a high cure rate for gastric cancer, the outcome of terminal surgical treatments remains discouraging. With lofty rates of recurrence, metastasis, mortality, and poor prognosis, novel approaches that induce cancer cell death demand urgent exploration.

Pyroptosis emerges as a compelling avenue among various cell death pathways 3. This intricate process hinges upon the activation of cysteine-dependent aspartate-specific proteases. Notably, mounting evidence suggests that the activation of NLRP3 inflammasome triggers cysteine (Caspase-1/4/11), ultimately culminating in pyroptosis induction. The hallmark characteristics of pyroptosis include the disruption of cellular membrane integrity, consequent release of inflammatory factors, and lactate dehydrogenase (LDH) 4, 5. The two predominant pathways, namely classical (Caspase-1 activated) and non-classical (Caspase-4/11 activated) avenues, shape the landscape of pyroptosis. Within the classical pathway, Caspase-1 assumes an activation state that propels Gasdermin D (GSDMD) cleavage, leading to its N-terminal liberation and the formation of cytosolic pores 6, 7. These pores facilitate the release of the inflammatory factors IL-18 and IL-1β. While recent studies have highlighted the crucial role of NLRP3-related pyroptosis in cancer therapy, the mechanistic intricacies surrounding pyroptosis in gastric cancer remain largely unexplored.

Reactive oxygen species (ROS), widely acknowledged as vital mediators of cell signaling, assume paramount importance. Under normal physiological conditions, ROS act as signaling molecules regulating cellular pathways essential for functional and homeostatic maintenance. Within the tumor environment, this regulatory capacity becomes particularly significant 8, 9. Recent research indicates that elevated ROS levels provoke oxidative stress, culminating in mitochondrial 10 and DNA damage 11, cell cycle arrest, and cellular senescence 12. Therefore, it is postulated that maintaining high ROS homeostasis can exert an anti-tumor effect.

Chinese herbal medicine has garnered extensive usage in the clinical treatment of gastric cancer. Aligned with the fundamental principles of Chinese medicine, which emphasize the spleen and stomach's pivotal role as the foundation of postnatal health and the production of qi and blood, Scutellaria Radix holds prominence. In adherence to the tenets of Chinese medicine theory, wherein the spleen detests dampness and favors dryness, the obstruction of dampness in the middle jiao disrupts the proper functioning of the spleen and stomach qi. This obstruction, coupled with the condensation of pathological factors like dampness, phlegm, stasis, and turbidity, ultimately leads to the development of gastric cancer. Consequently, Scutellaria Radix finds frequent application in clinical gastric cancer treatments due to its ability to clear internal heat, dry dampness, and facilitate detoxification. Baicalein (BA), a highly bioactive flavonoid extracted from Scutellaria baicalensis Georgi, forms a crucial component of drugs commonly employed in Chinese medicine for tumor treatment, including Scutellaria baicalensis Georgi, Pinellia ternata (Thunb.) Makino, and Achyranthes bidentata Blume. Numerous studies have substantiated the favorable anti-tumor effects of baicalin on various malignancies such as liver cancer 13, lung cancer 14, cervical cancer, colorectal cancer 15, and pancreatic cancer 16, with no significant cytotoxicity observed in normal cells. These findings highlight the promising future of baicalin as a potential anti-cancer substance for human therapeutics. However, the mechanism underlying baicalin's therapeutic effects on gastric cancer remains insufficiently elucidated, necessitating further investigation.

In this groundbreaking study, we demonstrate that BA holds the prowess to promote gastric cancer cell pyroptosis by activating the NF-κB signaling pathway. Remarkably, BA exhibits the ability to counteract the effects of the NLRP3 inhibitor (MCC950 Sodium) and synergistically enhances pyroptosis in a dose-dependent manner. Moreover, our prediction postulates that ROS serves as the upstream signal governing the NF-κB-NLRP3 signaling axis, thereby fueling the pyroptosis of gastric cancer cells.

## Materials and Methods

### Reagents

Baicalein (Purity ≥ 98% - Purchased from CHENGDU MUST BIO-TECHNOLOGY Co., Ltd), DMSO, CCK8 (Vazyme, China), NLRP3, Caspase-1, GSDMD, β-Actin, NF-κB, IL-18, IL-1β (Purchased from Abmart Shanghai Co., Ltd.).

### Cell and cultures

AGS (Human gastric gland carcinoma cell), nurtured in a conducive environment comprising 10% fetal bovine serum (ABW) supplemented with 1% streptomycin-penicillin 1640 medium (keyGEN BioTECH), finds solace within the confines of a temperature-controlled incubator set at a balmy 37 °C.

### Cell viability assay

Evaluating the vitality of cells assumes paramount importance, thus necessitating the performance of CCK8 assays to gauge their sensitivity to the drug at hand. Inoculating cells in 96-well plates at a density of 5*10^4 cells per well, we embark upon their nurturing journey. Following an overnight incubation period, the cells are subjected to varying concentrations of BA (6.25 uM/ml, 12.5 uM/ml, 25 uM/ml, 50 uM/ml, 100 uM/ml) over a span of 24 hours. To assess the cellular response, 10 ul of cck8 is introduced to each well, fostering a delicate symbiosis that endures a two-hour incubation at 37 °C, diligently protected from the prying grasp of light. Subsequent measurement of absorbance (OD) at 490 nm utilizing a Microplate Reader, with data representative of no less than three independent experiments.

### Molecular Docking

The crystal structures of proteins with high resolution and relatively intact structures were selected by the PDB database 17. Detailed data are as follows: Caspase-1 (ID: 6MFQ; 7AEH), Caspase-4 (ID: 5GPP; 5GPQ), Caspase-11 (ID: 3NIR; 3P4J), NLRP3 (ID: 3VWD), GSDMD (ID: 6BZ9), ASC (ID: 3VLN), IKB (ID: 4F0Z), IKKB (ID: 4KIK), NFκB (ID: 6P0Z). Prior to the docking extravaganza, a meticulous dance of pre-treatments involving dehydration and hydrogenation commences, orchestrated with the aid of Auto Dock Tools software. Engaging Autogrid in a simulated docking affair enables us to unearth binding energies that guide our discerning gaze towards molecules showcasing remarkable affinity. Molecules with strong binding energies were selected and visualized by using pymol software.

### Western blot

Cells were stimulated with different concentrations of baicalin for 24 h. Cellular extracted proteins were collected with the measurement of total protein concentration. The proteins were separated by electrophoresis with 10%, 12%, and 15% SDS-PAGE gel and transferred to PVDF membranes. After sealing the membranes with the skim milk powder, the membranes were incubated with primary antibodies (β-Actin, NLRP3, Caspase-1, GSDMD, IL-18, IL-1β, and NF-κB) at 4 °C overnight and secondary antibodies at room temperature for 2 hours. Visualize the strips using a luminescence imaging system (Chemi Doc XRS+).

### Quantitative real-time PCR (qRT-PCR) analysis

Total RNA was extracted from cells using HIScript II Q RT SuperMix for qPCR (+gDNA wiper) kit (Vazyme) and cDNA was synthesized using ChamQ Universal SYBR qPCR Master Mix kit (Vazyme). The detailed sequences of the primers used for RT-PCR are as follows:

Relative expression levels were analyzed by the 2-ΔΔCt method. PCR primers were synthesized by Shanghai Bioengineering Technology (Shanghai, China).

### Lactate dehydrogenase (LDH)release assay

An intriguing biochemical phenomenon, the release of lactate dehydrogenase (LDH), serves as a remarkable indicator of cell membrane rupture. To measure cellular LDH release, an LDH assay kit from the esteemed Nanjing Jiancheng Bioengineering Institute in China was employed. Tumor cells underwent stimulation with varying concentrations of BA (4 μM, 7 μM, 11 μM) for a duration of 24 hours. The inhibitor group received prior treatment with 20 μM MCC950 Sodium (MS) for a period of 2 hours, followed by incubation with diverse concentrations of BA for another 24 hours. The absorbance at 450 nm was meticulously detected utilizing a Microplate Reader.

### Network Pharmacology Analysis

Delving deeper into the intricate realm of pharmacology, therapeutic targets associated with baicalin were meticulously amassed through the remarkable Traditional Chinese Medicine Database and Analysis Platform (TCMSP) 18. In order to procure targets relevant to gastric cancer, the esteemed DrugBank database 19 and the DisGeNET v6.0 database 20 were diligently consulted. VENNY 2.1.0 online interactive software 21 was used to merge and de-duplicate the data. Further analysis was conducted employing the Metaspace database 22, specifically exploring the Kyoto Encyclopedia of Genes and Genomes (KEGG), Gene Ontology analysis- cellular component (GO-CC), and the multifaceted Gene Ontology biological process (GO-BP) and molecular function (GO-MF). The results were visualized by Cytospace to obtain the "Baicalin-Therapeutic Target-Pathway of Action" diagram.

### ROS detection

Cells were collected from each experimental group, and the positive control group was first stimulated with Rosup (100 μM) for 2 hours. Probes were loaded on cells using the Reative Oxygen Species Assay kit (Yeasen Biotechnolo (Shanghai) Co.,Ltd). Cell sorting by using flow cytometry.

### Statistical analysis

Statistical analysis was performed using GraphPad Prism 8 software, and comparison between two datasets demanded the meticulous employment of an independent samples t-test, where statistical significance was denoted by P<0.05. Furthermore, comparisons among multiple groups were astutely analyzed via Tukey's test, capitalizing on the foundation of one-way ANOVA.

## Results

### BA promotes gastric cancer cell pyroptosis

**Figure 1:**
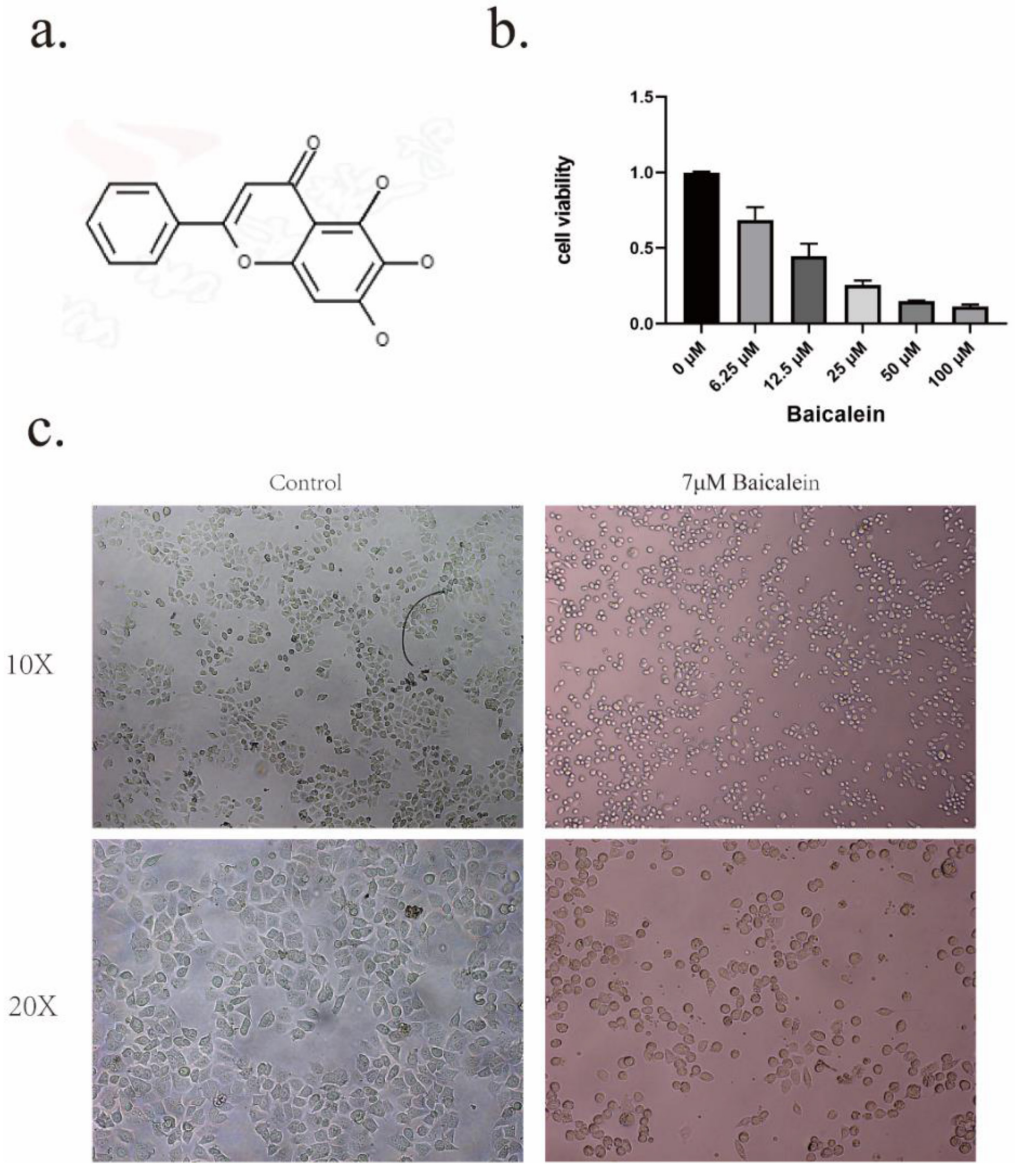
Inhibition of tumor cell activity by BA. a. Chemical structure formula of BA. b. CCK8 assay to evaluate the cytotoxic effect of BA on gastric cancer cells. c. Microscopic observation of cellular morphological changes following BA stimulation.

The investigation commenced by subjecting gastric cancer cells to a diverse range of BA (The structure of baicalein is shown in Figure 1a) concentrations (6.25uM, 12.5uM, 25uM, 50uM, 100uM) for a duration of 24 hours, as visually represented in Figure 1b. Notably, it revealing that the administration of BA at a concentration of 6.25uM already elicited a pronounced inhibition of cell growth within the gastric cancer cell population.To further explore the potency of baicalin's action on gastric cancer cells, the 24-hour IC50 value was determined to be 12.05uM. Based on this critical threshold, subsequent investigation focused on administering baicalin at the doses of 4uM, 7uM, and 11uM.

Microscopic examination provided further insights into the cellular dynamics following a 24-hour exposure to baicalin. Notably, the cell volume exhibited a significant reduction compared to the blank group, accompanied by a transformation in cell morphology from irregular to round-like, vacuoles emerged within the cellular milieu, and certain cell membranes underwent rupture, leading to the release of cellular contents (Figure 1c).

**Figure 2:**
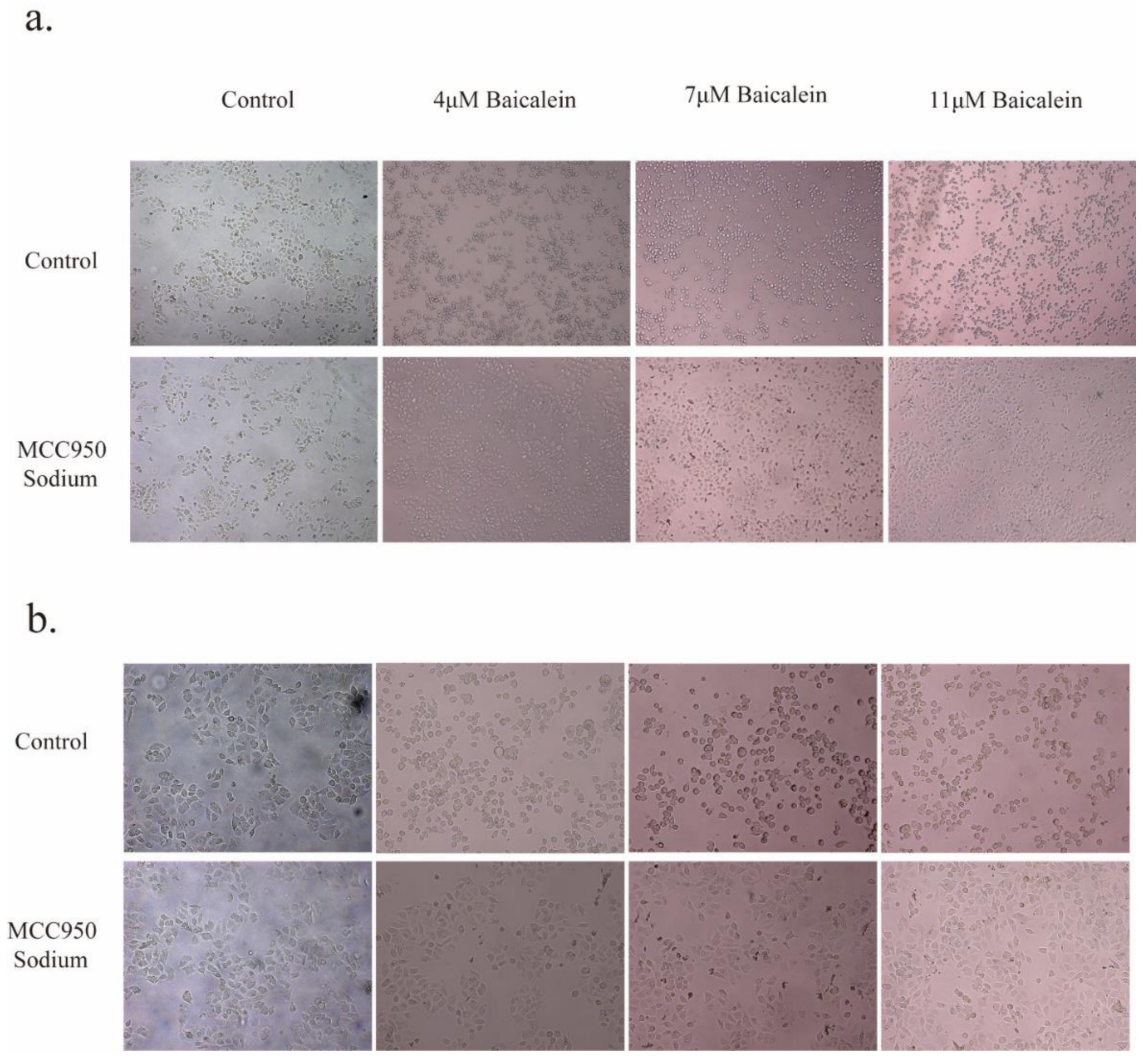
Effects of BA on the morphological changes and cell membrane integrity of gastric cancer cells. a. Microscopic observations were conducted at 10X magnifications after 24 hours of BA stimulation (4μM, 7μM, 11μM) in tumor cells. b. Microscopic observations were conducted at 20X magnifications after 24 hours of BA stimulation (4μM, 7μM, 11μM) in tumor cells.

Intriguingly, the inclusion of 20 μM MS failed to impede BA's capacity to induce tumor cell death and induce alterations in cellular morphology (Figure 2). Furthermore, an in-depth analysis of LDH levels, considered a consequential product of pyroptosis, revealed that the inhibition of NLRP3 expression by MS profoundly dampened the release of LDH. Conversely, baicalin exhibited a dose-dependent promotion of LDH release, surpassing the inhibitory effect induced by MS and resulting in higher levels of LDH release compared to baicalin alone (Figure 5a). These observations provide compelling evidence showcasing BA's ability to fuel the scorching of gastric cancer cells. It demonstrates that BA could promote gastric cancer cell pyroptosis.

### BA regulates NLRP3-induced pyroptosis in gastric cancer cells

It is widely acknowledged within scholarly circles that the modicum of docking energy exhibited by the protein-ligand complex and the therapeutic drug in question serves as a pivotal determinant of the binding conformation's stability. The underlying principle posits that a lower docking energy corresponds to heightened stability in the binding interactions. Specifically, docking energies below the threshold of -4.25 kJ are deemed indicative of binding activity between the protein-ligand complex and the drug. Energies falling below -7.0 kJ signify favorable docking outcomes, while those below -9.0 kJ herald robust and potent docking activities.

To gain insights into the mechanistic nuances underpinning Baicalin's actions, molecular docking simulations were scrupulously conducted. This astute undertaking aimed to pinpoint the precise target with which Baicalin intricately interacts. The ensuing results unveiled noteworthy associations, as NLRP3、Caspase-1、GSDMD, and ASC emerged as prominent entities that engaged in docking interactions with Baicalin. Notably, the binding affinity was most pronounced with NLRP3, boasting an exquisite binding energy of -9.2 kJ (Table 2).

**Figure 4:**
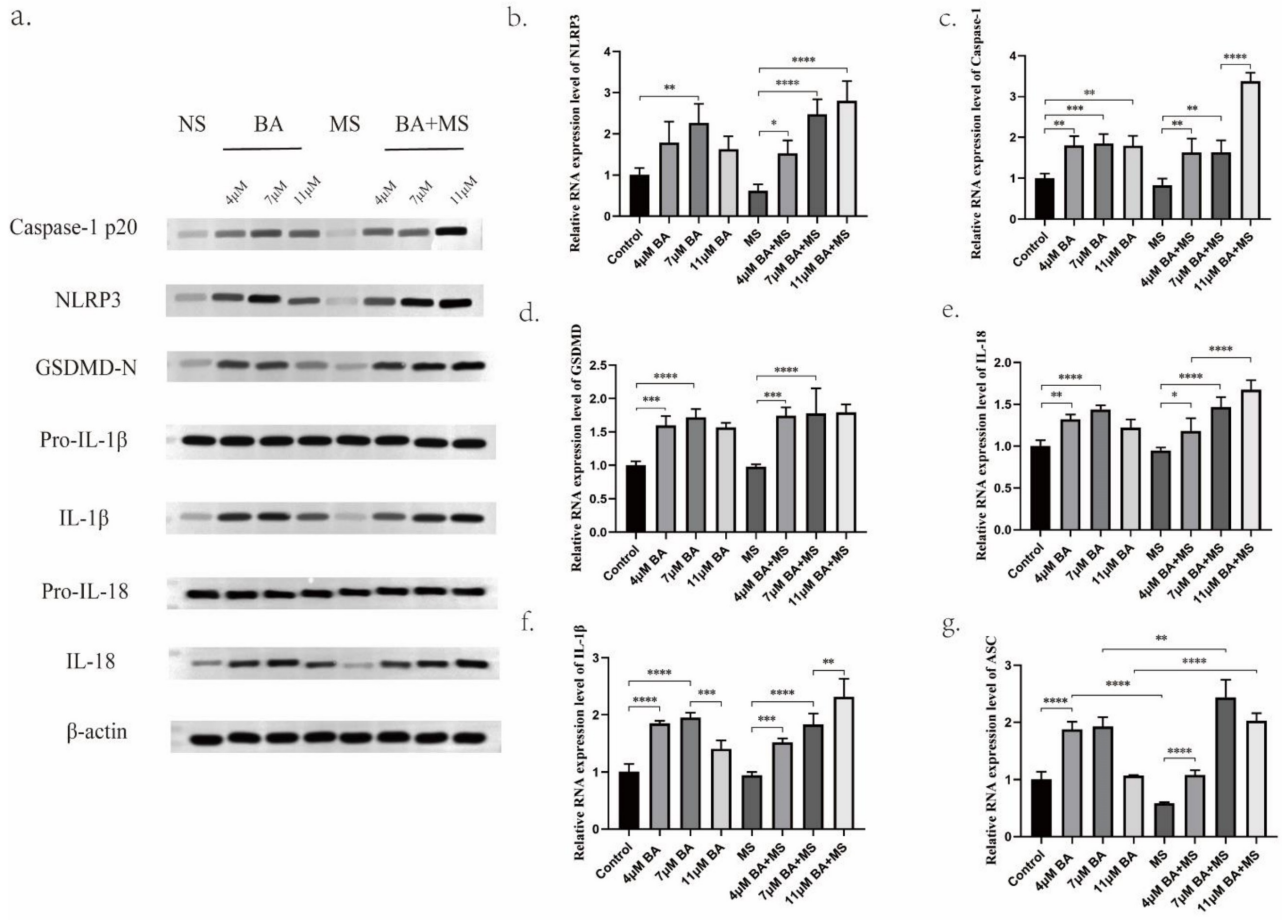
Promotion of gastric cancer cell pyroptosis by BA *in vitro*. a. Western blotting (WB) for protein level determination. b-g. Quantitative polymerase chain reaction (Q-PCR) to assess the mRNA levels of NLRP3, Caspase-1, GSDMD, IL-18, IL-1β, ASC.

**Figure 5:**
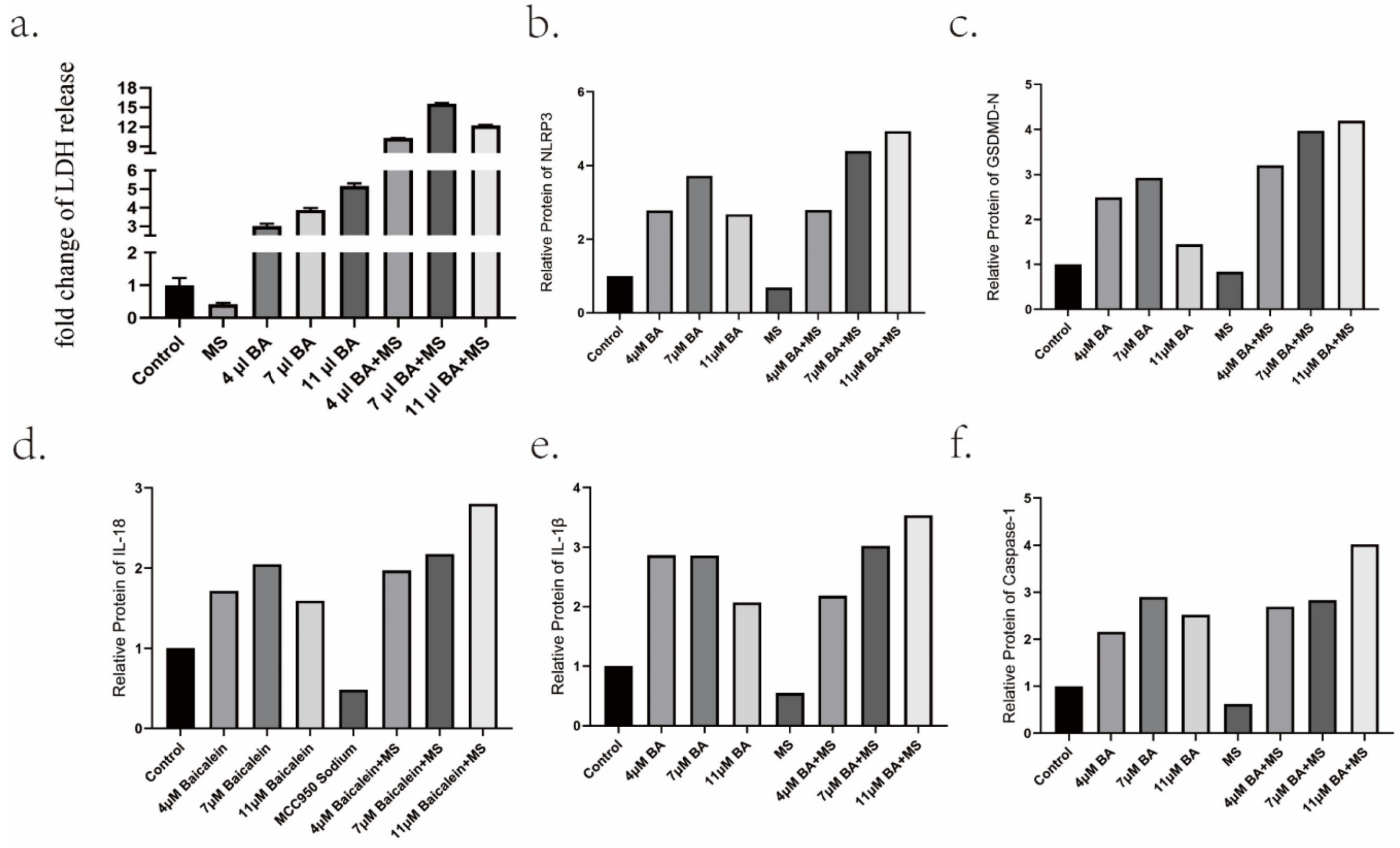
Promotion of gastric cancer cell pyroptosis by BA *in vitro*. a.Measurement of lactate dehydrogenase (LDH) release from baicalin-stimulated gastric cancer cells. b-f Relative protein levels of NLRP3, Caspase-1, GSDMD, IL-18, IL-1β.

The experimental analysis employing Western blotting (WB) and quantitative polymerase chain reaction (Q-PCR) methodologies has yielded substantive evidence demonstrating the capacity of Baicalin to modulate the expression levels of NLRP3, GSDMD-N, Caspase-1 P20, ASC protein, and mRNA. Notably, the findings reveal that the medium concentration of 7 μM Baicalin exerts a significantly more pronounced stimulatory effect on NLRP3 levels compared to the alternative concentration groups of 4 μM and 11 μM (Figure 4a,4b,5b). Conversely, the levels of Caspase-1-P20 (Figure 4a,4c,5f) and GSDMD-N (Figure 4a, 4d,5c) witness substantial increments upon Baicalin administration, albeit without displaying any overt dependence on dosage. Moreover, intriguing dose-dependent variations are evident in the ASC levels stimulated by Baicalin treatment. Specifically, lower and medium concentrations of Baicalin provoke significant elevations in ASC levels, while higher concentrations thereof elicit negligible effects on ASC expression (Figure 4g). In addition, the consequential evaluation of pyroptosis products, namely IL-18 and IL-1β, attests to the ability of Baicalin to augment their levels. The experimental outcomes reveal that Baicalin induces a robust increase in IL-18 (Figure 4a, 4e, 5d) and IL-1β (Figure 4a, 4f, 5e) levels, with the medium concentration group (7 μM) eliciting the most prominent effect.

Furthermore, the effects of Baicalin under the pretreatment of MS were investigated, yielding observations. Remarkably, Baicalin exhibits a dose-dependent reversal of the inhibitory influence exerted by MS on NLRP3 expression levels in gastric cancer cells. Baicalin demonstrates augmented NLRP3, Caspase-1 P20, and ASC expression levels when subjected to MS pretreatment. Upon careful consideration of concentration factors, it is evident that under MS pretreatment, baicalin exhibited a stronger stimulatory effect on the expression of NLRP3 in the high concentration group (11 μM) as compared to the low concentration group (4 μM) and medium concentration group (7 μM). Moreover, all concentrations of Baicalin effectively counteract the inhibitory impact of MS on ASC in gastric cancer cells. Subsequent to subjecting scorched products IL-18 and IL-1β to pre-treatment with MS, notable insights of discernible nature arise. Specifically, an examination of the levels of IL-18 and IL-1β within the low and medium concentration groups reveals a lack of significant distinctions. However, in marked contrast, the high concentration group exhibits a substantial augmentation in both IL-18 and IL-1β levels, surpassing those induced by equivalent doses of Baicalin administered independently. This striking divergence in the response underscores the intricate interplay between MS pretreatment and Baicalin administration, thereby accentuating potential regulatory mechanisms underlying the modulation of these pyroptosis mediators.

### BA affects NLRP3- caspase-1 via NF-κB to promote gastric cancer cell Pyroptosis

Baicalein, a flavonoid compound, was subjected to comprehensive evaluation using the Traditional Chinese Medicine Systems Pharmacology (TCMSP) database 18, which provided insightful information regarding its pharmacological attributes. Considerable drug similarity (DL: 0.21) and favorable oral absorption (OB: 33.52%) were uncovered, suggesting its potential as a therapeutic agent. In light of this promising discovery, thorough investigations were conducted to elucidate the intricate molecular interactions underlying BA's effects.

**Figure 6:**
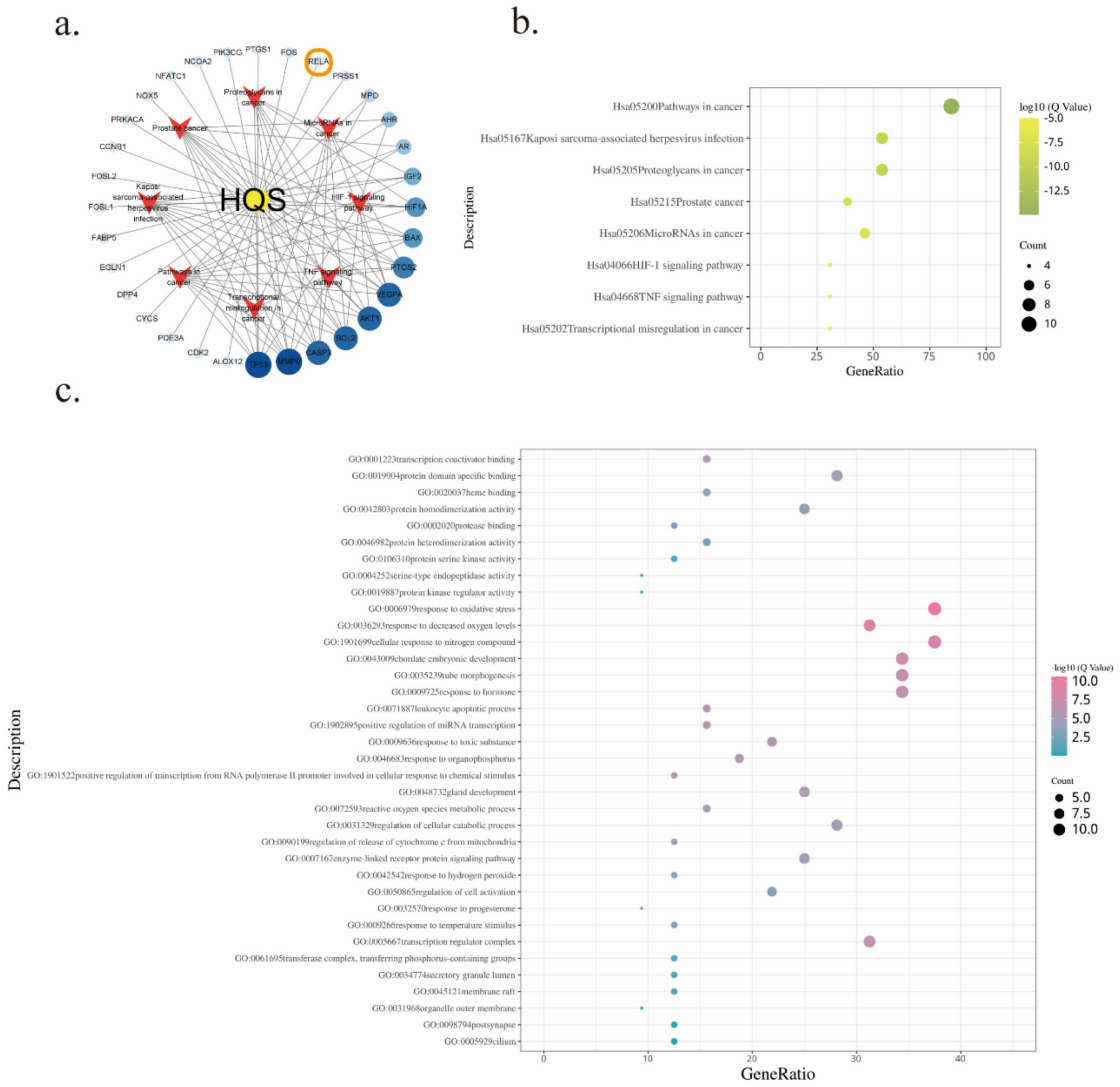
Bioinformatics analysis of baicalin in gastric cancer treatment.a. Network diagram depicting the target-pathway interactions of baicalin in gastric cancer.b. KEGG enrichment analysis of baicalin's therapeutic targets in gastric cancer.c. GO enrichment analysis of baicalin's targeted actions in gastric cancer treatment.

To discern the specific targets pertinent to gastric cancer, an amalgamation of data from the DisGeNET and GeneCards databases was undertaken, employing rigorous de-duplication procedures. Resulted in the identification of 32 unique co-action targets shared by BA and gastric cancer compounds (Figure 6a). These co-action targets were then subjected to comprehensive functional enrichment analyses utilizing the Meatscape database 22.

The outcome of these analyses unveiled compelling insights into the regulatory processes modulated by BA. Notably, among the enriched results derived from KEGG (Table 3) and GO (Table 4) analyses, the TNF signaling pathway emerged as a prominent pathway under the influence of BA (Figure 6b). This finding underscores the regulatory potential of BA in orchestrating crucial cellular responses mediated by tumor necrosis factor (TNF). Further exploration into the biological processes impacted by BA illuminated several noteworthy facets. Response to oxidative stress, reactive oxygen species metabolic process, and regulation of cellular catabolic process were among the intricate molecular cascades influenced by BA (Figure 6c). These findings collectively shed light on the diverse regulatory mechanisms through which BA exerts its influence within the complex milieu of gastric cancer cells.

**Figure 7:**
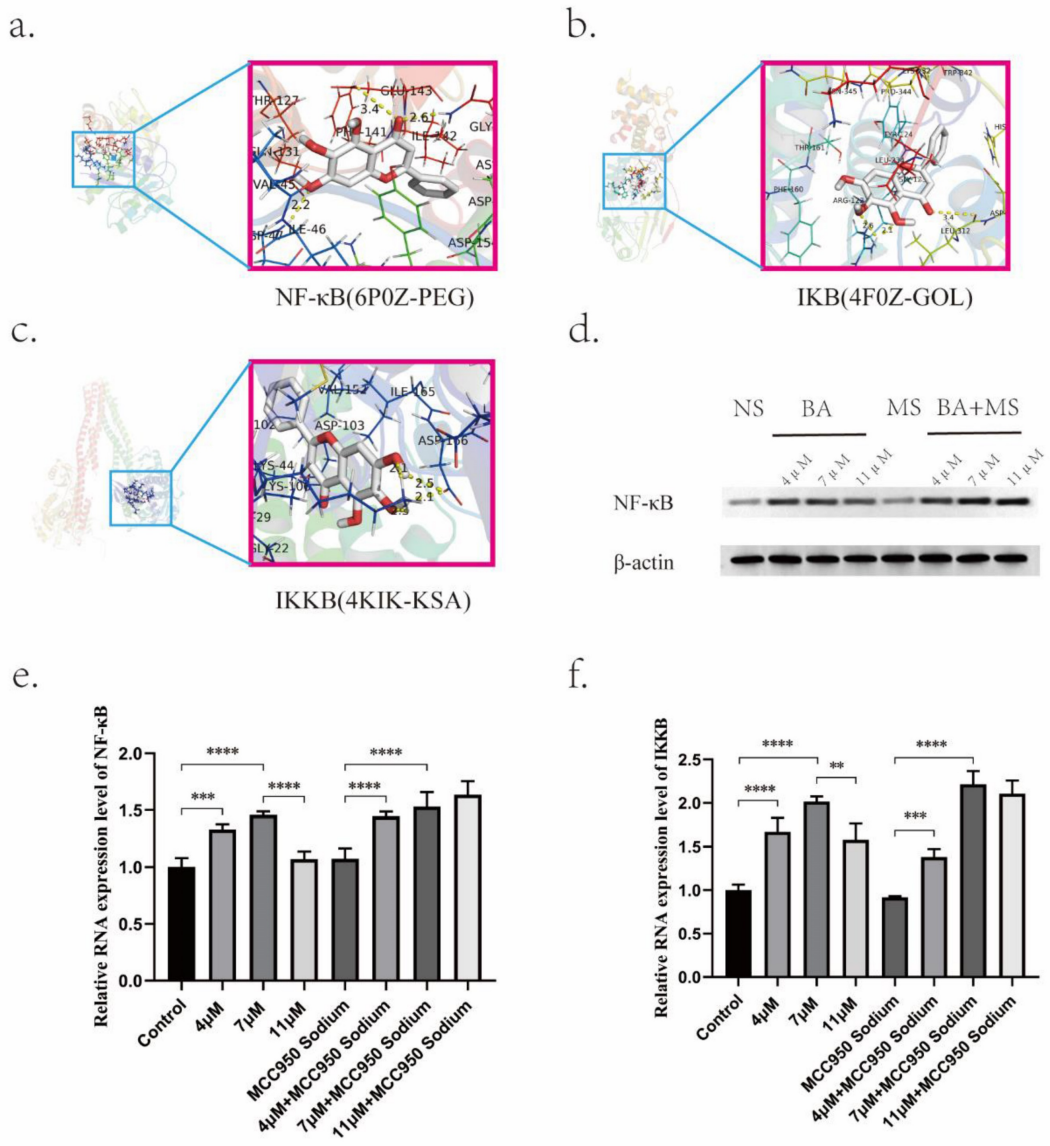
Effects of BA on the expression of key proteins. a-c. Molecular docking of BA with NF-κB, IKB, and IKKB. d. Western blot analysis to evaluate the protein expression levels of NF-κB under BA stimulation. e-f. q-PCR detection of mRNA expression levels of NF-κB and IKKB under BA stimulation.

Of particular significance was the identification of RELA protein as a key player associated with BA's effects. RELA, a pivotal component of the nuclear factor kappa B (NF-κB) pathway, displayed a robust association with BA based on docking studies involving NF-κB, IKB, and IKKB (Figure 7a,7b,7c). Molecular docking simulations revealed the strong affinities between BA and NF-κB (Table 5). This interaction underscores the potential role of BA in modulating NLRP3-caspase-1 signaling, thereby facilitating the release of inflammatory factors during tumor cell pyroptosis via the NF-κB pathway. Moreover, investigations into the concentration-dependent effects of BA on NF-κB (p65) mRNA levels uncovered intriguing stage-specific discrepancies (Figure 7d). Notably, low (4 μM) and medium concentrations (7 μM) of BA elicited dose-dependent increases in NF-κB (RELA) and IKKB mRNA levels. In contrast, the excessively high concentration of BA (11 μM) manifested reduced expression levels compared to the low- and medium-dose groups (Figure 7e,7f).

### BA promotes a dramatic increase in ROS levels in gastric cancer cells

**Figure 8:**
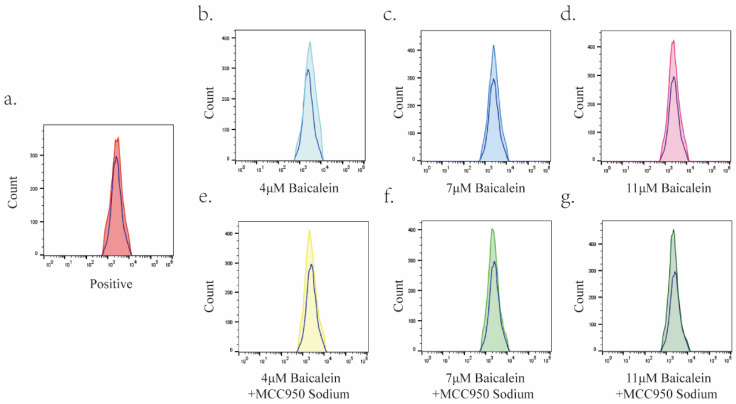
Upregulation of Reactive Oxygen Species (ROS) Levels in Gastric Cancer Cells by BA. In this figure, the blue hollow peaks represent the blank group, while the solid colored peaks correspond to the positive control group, BA dose group, and MS+BA dose group. Specifically: a: Illustrates the peak overlap between the positive control group and the blank group. b-d: Depict the peak overlap between the 4μM, 7μM, and 11μM BA groups and the blank group. e-g: Highlight the peak overlap plots of the 4μM, 7μM, and 11μM BA groups with the blank group under MS pretreatment.

The impact of different concentrations of BA (4 μM, 7 μM, and 11 μM) on reactive oxygen species (ROS) levels within AGS cells was investigated over a 24-hour incubation period. Flow cytometry analysis was employed to quantify the changes in ROS levels under each condition (Figure 8). Notably, BA treatment resulted in a significant elevation in ROS levels within gastric cancer cells when compared to the positive control group (Rosup). Furthermore, no noticeable differences in ROS levels were observed among the various BA dose groups.

## Discussion

The intricate and multifaceted biological progression of gastric cancer (GC) is governed by a plethora of molecular and cellular signaling pathways. In recent years, the incorporation of Chinese medicine into the domain of antitumor therapeutics has garnered considerable attention due to its demonstrated clinical efficacy. Of notable significance is the emergence of Scutellaria baicalensis as a natural remedy with vast prospects, as its potent antitumor effects have been corroborated by clinical investigations 23-25. This compelling observation has propelled our research team to delve deeper into unraveling the intrinsic mechanisms underpinning the antitumor activity of the principal component of Scutellaria baicalensis, namely baicalin.

Our investigation commences with an integrative approach employing molecular docking and meticulous microscopic morphology assessments to postulate the impact of BA on Caspase-1, thereby instigating pyroptotic cell death in gastric cancer cells. Subsequently, leveraging the power of Western blotting (WB) and quantitative polymerase chain reaction (qPCR) techniques, we meticulously scrutinize the expression profiles of pivotal components including NLRP3, GSDMD-N, ASC, Caspase-1, IL-18, and IL-1β, culminating in a demonstration of the dose-dependent promotion of AGS cell pyroptosis by BA. NLRP3 inflammasomes, comprising sensor proteins such as NLRP3, adaptor protein ASC, and effector protein caspase-1 26, represent indispensable cytoplasmic protein complexes orchestrating inflammatory responses 27, 28. Perturbed activation of NLRP3 can perpetuate chronic inflammation, thus significantly influencing the pathophysiology of inflammation-related diseases, while concurrently exhibiting profound tumor-suppressive properties 27. To gain further insights into the pro-pyroptotic mechanistic intricacies pertaining to BA, the NLRP3 inhibitor MS is employed, revealing an unequivocal reversal of the inhibitory effects imposed by this inhibitor, thereby conferring invaluable understanding into BA's potential in counteracting NLRP3 inhibitors. Collectively, these findings lend credence to the ability of BA to effectively activate NLRP3 inflammasomes, ultimately culminating in pyroptsis in gastric cancer cells.

By means of initial bioinformatics analyses, we present compelling evidence concerning BA's regulatory influence over the RELA gene expression, and reactive oxygen species (ROS) levels. Subsequently, through employment of WB and qPCR assays, we substantiate BA's ability to modulate NF-κB signaling.

Cancer cells, notorious for their relentless pursuit of survival mechanisms, have developed intricate strategies to counteract the detrimental effects of ROS accumulation. Perturbations in normal redox balance due to dysregulated ROS production can induce oxidative stress, which serves as a double-edged sword, exacerbating cellular damage while concurrently awakening pro-survival pathways in cancer cells. Hence, the ability to manipulate redox dynamics and modulate ROS levels selectively assumes paramount importance in devising innovative therapeutic interventions targeting cancer.

In conclusion, our comprehensive study explores the mechanisms underlying BA's antitumor effects in the context of gastric cancer. BA exhibits the capability to induce pyroptosis in gastric cancer cells, primarily through the activation of NLRP3 inflammasomes. Additionally, BA showcases regulatory potential on the RELA gene expression, and ROS levels, thereby influencing NF-κB signaling.

## Conclusion

The findings obtained from the experimental investigation unequivocally demonstrate the capacity of BA, a naturally occurring compound, to elicit pyroptotic cell death of gastric cancer cells. This is orchestrated through the induction of NLRP3 inflammasome activation, thereby underscoring the fundamental significance bestowed upon NLRP3 in mediating the BA-induced cascade culminating in cell pyroptosis.Moreover, the data shed light on a positive correlation between the NF-κB signaling pathway and the occurrence of pyroptosis events, emphasizing the interplay between these molecular cascades in dictating the fate of the affected cells. Further elucidation of the mechanisms underlying BA's anticancer effects revealed its ability to stimulate elevated levels of reactive oxygen species (ROS), thus unveiling an additional facet of its therapeutic potential in combating gastric malignancies.

## Figures and Tables

**Figure 3:**
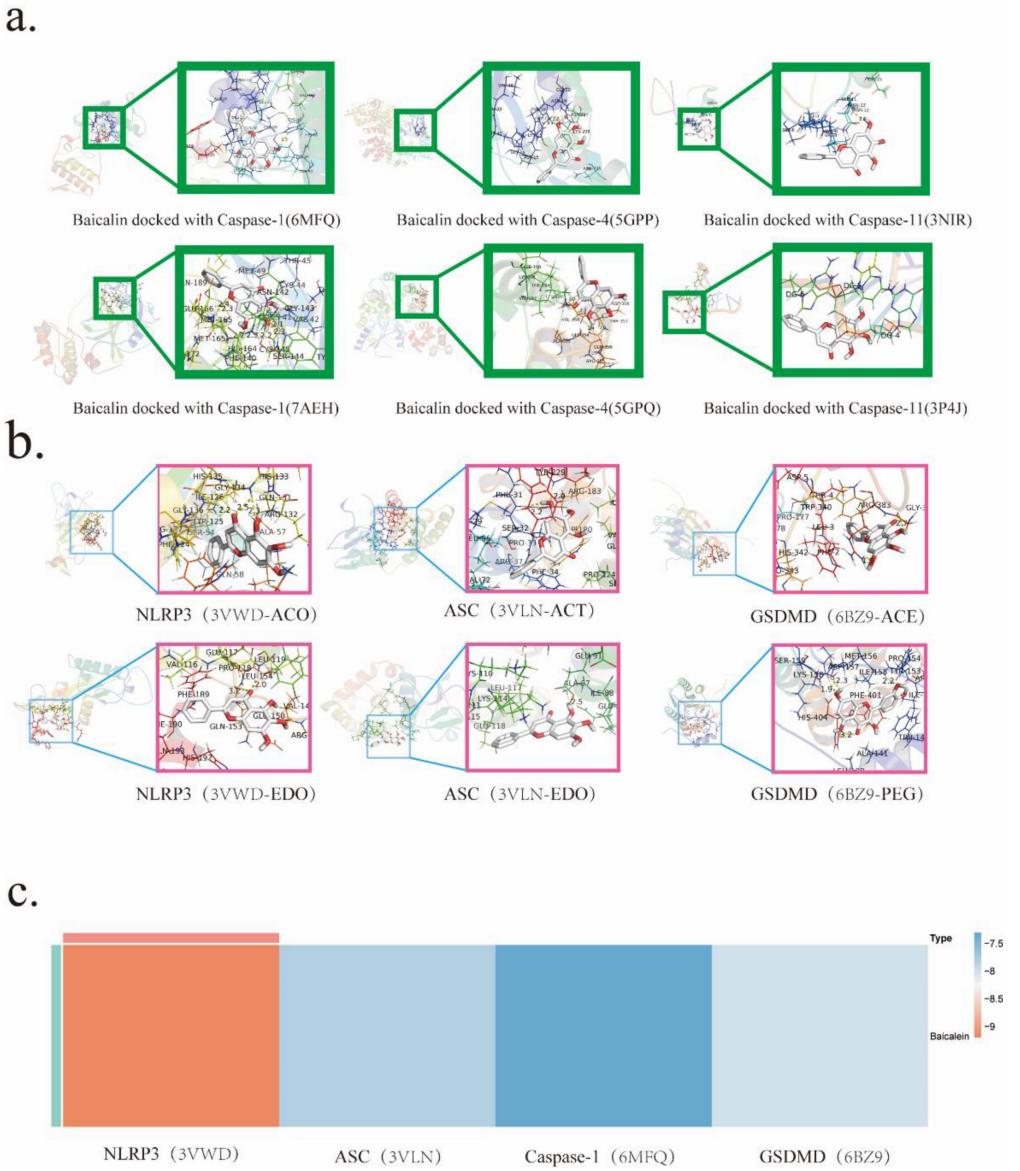
Molecular docking analysis predicting BA's target interactions.a. Molecular docking of BA with Caspase-1/4/11. b. Molecular docking of BA with NLRP3, ASC, GSDMD.c. Heat map visualizing the results of BA's molecular docking with NLRP3、ASC、Caspase-1 and GSDMD.

**Figure 9:**
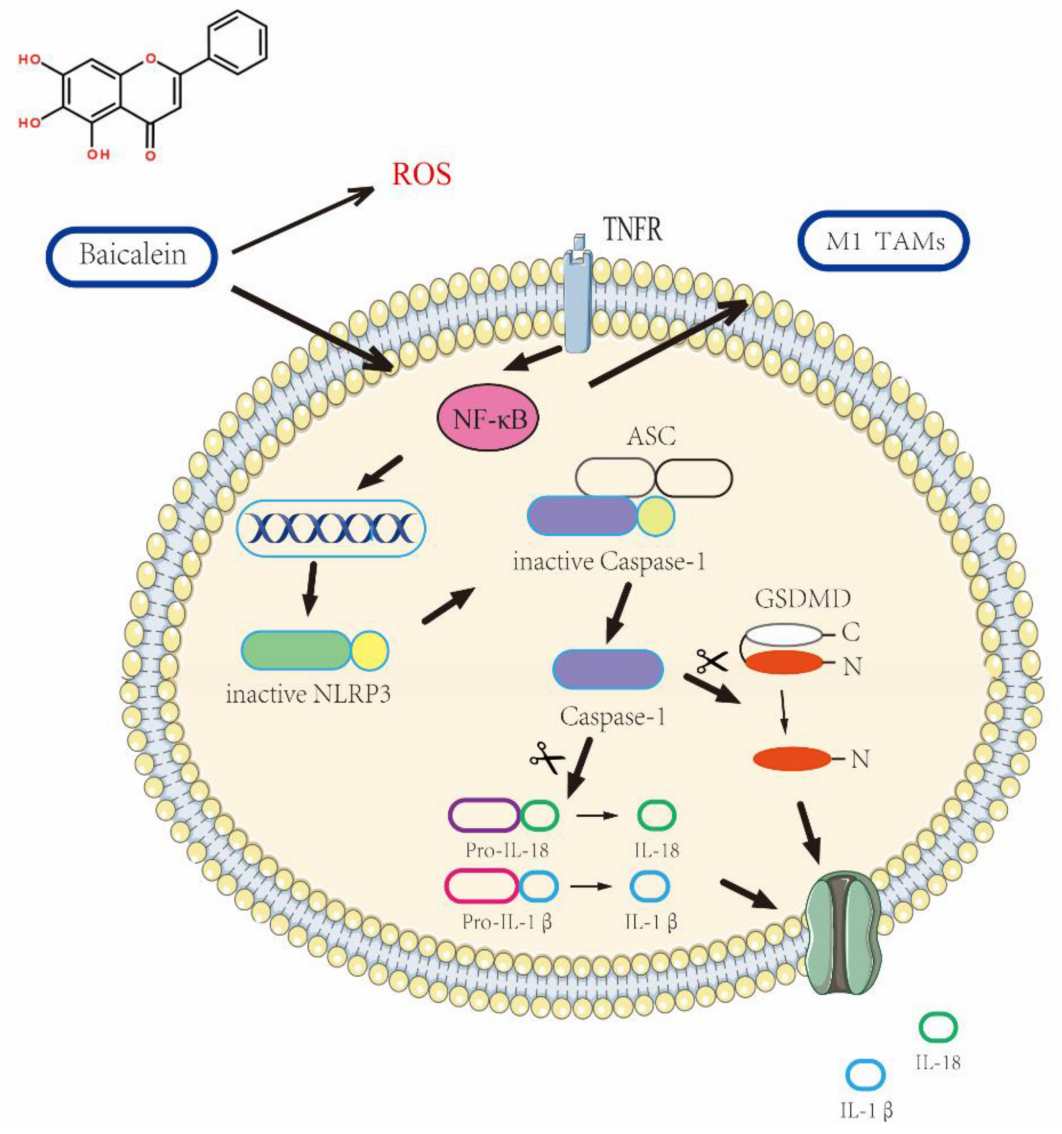
Elucidation of the Mechanism Underlying BA-Induced pyroptosis in gastric cancer Cells.

**Table 1:** Primer sequences

Primer	Primer Sequences	
GAPDH	Forward	5'- AGAACATCATCCCTGCCTCTACTGG-3'
	Reverse	5'- CGCCTGCTTCACCACCTTCTTG-3'
Caspase-1	Forward	5'- TCCTCAGGCTCAGAAGGGAATGTC -3'
	Reverse	5'- GTGCGGCTTGACTTGTCCATTATTG -3'
NLRP3	Forward	5'- GCCAGGAAGACAGCATTGAAGAGG -3'
	Reverse	5'- AGTCGTGTGTAGCGTTTGTTGAGG -3'
GSDMD	Forward	5'- ACAGCTCCAGCACCTCAATGAATG -3'
	Reverse	5'- GCACCTCAGTCACCACGTACAC -3'
NF-κB	Forward	5'- TCTCTCGCCTGCCTCCACAAG -3'
	Reverse	5'- GATGTCTCCACGCCGCTGTC -3'
IKKB	Forward	5'- ACAGAGGAGGGACAGAAAGGGTATC -3'
	Reverse	5'- CACGCTTGAGCAGGAATGGAGAC-3'
ASC	Forward	5'- GCCCACCAACCCAAGCAAGATG -3'
	Reverse	5'- CTCCGCTCCAGGTCCTCCAC-3'

**Table 2:** Molecular docking energy

GENE	Ligand	Docking energy
Caspase-1	6MFQ	-7.8KJ/mol
	7AEH	-5.7KJ/mol
Caspase-4	5GPP	-5.3KJ/mol
	5GPQ	-5.1KJ/mol
Caspase-11	3NIR	-5.4KJ/mol
	3P4J	-3.5KJ/mol
NLRP3	3VWD(ACO)	-9.2KJ/mol
	3VWD(EDO)	-6.9KJ/mol
ASC	3VLN(GOL)	-6.4KJ/mol
	3VLN(ACT)	-7.3KJ/mol
GSDMD	6B29(ACE)	-4.3KJ/mol
	6B29(DEC)	-8.0KJ/mol

**Table 3:** KEGG pathway analysis of the effects of BA on gastric cancer cells

SampleGroup	Qvalue	Count	Description
KEGG Pathway	1.17E-15	11	hsa05200Pathways in cancer
KEGG Pathway	9.77E-11	7	hsa05167Kaposi sarcoma-associated herpesvirus infection
KEGG Pathway	9.77E-11	7	hsa05205Proteoglycans in cancer
KEGG Pathway	1.29E-08	5	hsa05215Prostate cancer
KEGG Pathway	4.79E-08	6	hsa05206MicroRNAs in cancer
KEGG Pathway	1.62E-06	4	hsa04066HIF-1 signaling pathway
KEGG Pathway	1.74E-06	4	hsa04668TNF signaling pathway
KEGG Pathway	1.1E-05	4	hsa05202Transcriptional misregulation in cancer

**Table 4:** GO pathway analysis of the effects of BA on gastric cancer cells

Group	%	Qvalue	Count	Description
GO-MF	15.62	2.63E-06	5	Transcription coactivator binding
GO-MF	28.12	1.82E-05	9	Protein domain specific binding
GO-MF	15.62	0.000174	5	Heme binding
GO-MF	25	0.000204	8	Protein homodimerization activity
GO-MF	12.5	0.00309	4	Protease binding
GO-MF	15.62	0.005248	5	Protein heterodimerization activity
GO-MF	12.5	0.085114	4	Protein serine kinase activity
GO-MF	9.38	0.120226	3	Serine-type endopeptidase activity
GO-MF	9.38	0.194984	3	Protein kinase regulator activity
GO-BP	37.5	2.4E-11	12	Response to oxidative stress
GO-BP	31.25	1.2E-09	10	Response to decreased oxygen levels
GO-BP	37.5	2.45E-09	12	Cellular response to nitrogen compound
GO-BP	34.38	3.31E-08	11	Chordate embryonic development
GO-BP	34.38	4.57E-08	11	Tube morphogenesis
GO-BP	34.38	1.35E-07	11	Response to hormone
GO-BP	15.62	4.57E-07	5	Leukocyte apoptotic process
GO-BP	15.62	7.41E-07	5	Positive regulation of mirna transcription
GO-BP	21.88	1.58E-06	7	Response to toxic substance
GO-BP	18.75	1.78E-06	6	Response to organophosphorus
GO-BP	12.5	2.4E-06	4	Positive regulation of transcription from RNA polymerase II promoter involved in cellular response to chemical stimulus
GO-BP	25	3.55E-06	8	Gland development
GO-BP	15.62	1.86E-05	5	Reactive oxygen species metabolic process
GO-BP	28.12	2.19E-05	9	Regulation of cellular catabolic process
GO-BP	12.5	3.63E-05	4	Regulation of release of cytochrome c from mitochondria
GO-BP	25	4.79E-05	8	Enzyme-linked receptor protein signaling pathway
GO-BP	12.5	0.000468	4	Response to hydrogen peroxide
GO-BP	21.88	0.000759	7	Regulation of cell activation
GO-BP	9.38	0.000776	3	Response to progesterone
GO-BP	12.5	0.002089	4	Response to temperature stimulus
GO-CC	31.25	1.55E-07	10	Transcription regulator complex
GO-CC	12.5	0.079433	4	Transferase complex, transferring phosphorus-containing groups
GO-CC	12.5	0.079433	4	Secretory granule lumen
GO-CC	12.5	0.079433	4	Membrane raft
GO-CC	9.38	0.323594	3	Organelle outer membrane
GO-CC	12.5	0.57544	4	Postsynapse
GO-CC	12.5	0.912011	4	Cilium

**Table 5:** Molecular docking energy

Gene	Ligand	Docking energy
Nf-κb	6p0z(peg)	-9.0kj/mol
Ikb	4f0z(gol)	-7.4kj/mol
Ikkb	4kik(ksa)	-9.5kj/mol
